# A network-based biomarker approach for molecular investigation and diagnosis of lung cancer

**DOI:** 10.1186/1755-8794-4-2

**Published:** 2011-01-06

**Authors:** Yu-Chao Wang, Bor-Sen Chen

**Affiliations:** 1 Laboratory of Control and Systems Biology, Department of Electrical Engineering, National Tsing Hua University, Hsinchu 30013, Taiwan

## Abstract

**Background:**

Lung cancer is the leading cause of cancer deaths worldwide. Many studies have investigated the carcinogenic process and identified the biomarkers for signature classification. However, based on the research dedicated to this field, there is no highly sensitive network-based method for carcinogenesis characterization and diagnosis from the systems perspective.

**Methods:**

In this study, a systems biology approach integrating microarray gene expression profiles and protein-protein interaction information was proposed to develop a network-based biomarker for molecular investigation into the network mechanism of lung carcinogenesis and diagnosis of lung cancer. The network-based biomarker consists of two protein association networks constructed for cancer samples and non-cancer samples.

**Results:**

Based on the network-based biomarker, a total of 40 significant proteins in lung carcinogenesis were identified with carcinogenesis relevance values (CRVs). In addition, the network-based biomarker, acting as the screening test, proved to be effective in diagnosing smokers with signs of lung cancer.

**Conclusions:**

A network-based biomarker using constructed protein association networks is a useful tool to highlight the pathways and mechanisms of the lung carcinogenic process and, more importantly, provides potential therapeutic targets to combat cancer.

## Background

Cancer, the complex disease of uncontrolled cell growth, is one of the leading causes of human death worldwide and the deaths from cancer are projected to continue rising [1,2]. Among all types of cancer, the most commonly diagnosed, as well as the most common cause of cancer deaths, is lung cancer, with a mortality rate as high as 80-85% within 5 years [1,3]. Lung cancer is categorized into two main types: small cell lung carcinoma (SCLC) and non-small cell lung carcinoma (NSCLC). NSCLCs are subcategorized into three main subtypes: squamous cell carcinoma, adenocarcinoma, and large cell carcinoma [4]. Previous research has shown that these major histological types of lung cancer are associated with cigarette smoking [5]. In light of this, much research has been devoted to investigating the molecular alterations which ensued from cigarette smoking and the mechanism that links cigarette smoking to lung cancer. Spira *et al*. used DNA microarray to compare the gene expressions of large-airway epithelial cells from nonsmokers and smokers, and to determine how cigarette smoking alters the transcriptome [6]. Hecht indicated that many tobacco smoke carcinogens, such as polycyclic aromatic hydrocarbons and nicotine-derived nitrosamine ketone are predominant inducers of lung cancer [7]. Recently, Takahashi *et al*. showed that induction of IKKβ- and JNK1-dependent inflammation is likely to be an important contributor to the tumor-promoting activity of tobacco smoke [8].

In addition to the investigation on carcinogenesis, many studies identified cancer biomarkers through analysis of genome-wide expression profiles [9,10]. The biomarkers are used either as a diagnostic evaluation to determine the health of a patient with or without the cancer, or as a prognostic indicator to determine the patient's prognosis. Spira *et al*. used gene expression profiles of samples from lung cancer patients to identify an 80-gene biomarker that distinguished apparent differences between smokers with and without lung cancer [3]. Because there is no effective screening tool for diagnosing lung cancer at an early stage, the 80-gene biomarker could make a beneficial contribution to minimizing high mortality rates by providing a better prognosis [3,11]. However, the biomarker identification method, which strictly uses gene expression profiles, cannot show how the different genes within the biomarker gene set are related to each other, i.e., the biomarkers are not identified from the systems perspective. Further, the gene lists obtained for similarly diagnosed patients by different research groups differ widely and share few common genes [12].

Due to these kinds of limitations and the widely accepted opinion that cancer is a disease of pathways [13,14], both protein-protein interaction (PPI) and pathway information are integrated for biomarker identification. Chuang *et al*. developed a protein-network-based approach that identifies biomarkers not as individual genes but as sub-networks extracted from protein interaction databases. They showed that the sub-network classification is highly accurate in signature discrimination and provide an accurate account of the network structure [15]. Many other network-based approaches for prioritizing disease genes and protein interaction subnetworks that are discriminative of disease signature have been developed [16-19]. The dynamic structure of the human protein interaction network has recently been examined to aid in predicting breast cancer prognosis, suggesting that network modularity might be a defining feature of tumor phenotype [20].

Network analysis has shown that under different cellular states or in response to diverse stimuli, transcription factors alter their interactions to regulate different genes, thereby rewiring the network [21]. The same situation occurs with protein interaction networks [20,22]. Motivated by the dynamic structure of the human protein interaction network and the observation that interacting proteins tend to result in similar disease phenotypes when dysregulated [23], we developed a computational framework to construct the network-based biomarker for molecular investigation and diagnosis of lung cancer. The network-based biomarker consisted of two protein association networks for cancer and non-cancer smokers. Based on the concept of network comparison [24], 40 significant proteins that play potentially important roles in lung carcinogenesis were identified. The network-based biomarker is a useful tool for further distinguishing the presence of cancer in smokers by similarity measurement of molecular patterns. Hopefully, the proposed method can aid in further understanding lung carcinogenesis and providing potential drug targets for humans to combat lung cancer.

## Methods

### Overview of the network-based biomarker approach for lung cancer investigation

**Figure 1:**
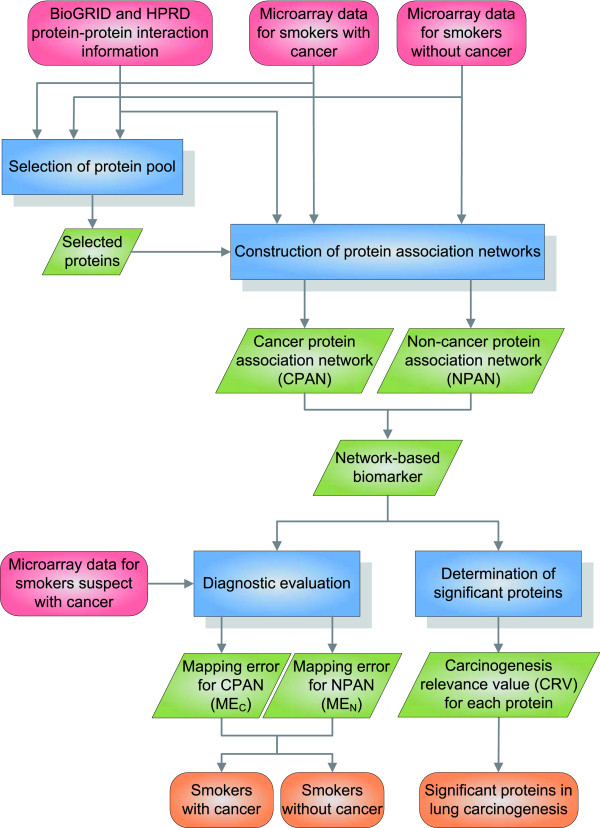
**The flowchart of constructing the network-based biomarker for lung cancer investigation and diagnosis**. The figure indicates the flowchart of the proposed method. Red represents the data needed. Blue denotes the processing steps of the approach. Green represents the processed results of each step and orange denotes the overall results from the entire method. In summary, two kinds of data, microarray data and PPI information, are needed for the proposed method. These data are used for protein pool selection, and then the selected proteins and the input data are used for protein association network construction, resulting in cancer protein association network (CPAN) and non-cancer protein association network (NPAN). The two constructed protein association networks form the overall network-based biomarker, which can be used for either determination of significant proteins or diagnostic evaluation. With the help of the network-based biomarker, carcinogenesis relevance value (CRV) is computed for each protein, and significant proteins in lung carcinogenesis are determined based on the CRVs. These significant proteins provide targets for further characterization. On the other hand, given the microarray data for smokers suspect of cancer, mapping errors for CPAN and NPAN can be computed, respectively, which help diagnose the smokers with cancer or without cancer.

The overall flowchart of the proposed network-based biomarker approach is shown in Figure 1. Our goal is to investigate lung cancer by the construction of network-based biomarkers composed of protein association networks for smokers with and without cancer. Microarray gene expression profiles of patient samples and protein-protein interaction information were integrated for protein selection and network construction. Two protein association networks with quantitative protein association abilities for cancer and non-cancer smokers were constructed. By comparing two protein association networks within the network-based biomarker, a carcinogenesis relevance value (CRV) was computed to correlate proteins with the level of lung carcinogenesis. A higher score suggests the particular protein plays a more critical role in lung carcinogenesis. A set of significant proteins was selected based on the CRV for each protein and the statistical assessment. Further, using microarray data for smokers suspected of having cancer, it is possible to compute mapping errors for further diagnostic evaluation of smokers with or without cancer.

### Data selection and preprocessing

In this study, two kinds of data, microarray gene expression profile and protein-protein interaction information, were integrated. The microarray data were downloaded from the GEO database http://www.ncbi.nlm.nih.gov/geo/ (accession number GSE4115). Spira *et al*. performed gene expression profiling in histologically normal large-airway epithelial cells obtained by bronchoscopy of current and former smokers. The health of each individual was recorded following the bronchoscopy until a final diagnosis of either presence or absence of lung cancer [3]. Data were collected from a total pool of 187 subjects and divided into primary and prospective data sets: 79 and 73 smokers with and without lung cancer respectively in the primary data set; 18 and 17 smokers with and without lung cancer respectively in the prospective data set. The primary data set was used for network-based biomarker construction and the prospective data set was used for diagnostic evaluation. Protein-protein interaction (PPI) data were extracted from the Biological General Repository for Interaction Datasets (BioGRID) http://thebiogrid.org/ and the Human Protein Reference Database (HPRD) http://www.hprd.org/. The BioGRID database was developed to house and distribute collections of protein and genetic interactions from major model organism species. BioGRID currently contains over 340,000 interactions derived from both high-throughput studies and conventional focused studies [25]. The HPRD is a database that integrates a wealth of information relevant to the human proteome, including protein-protein interactions, post-translational modifications, disease associations, and tissue expression [26]. Prior to further processing, the gene expression value *g_ij _*is normalized to z-transformed scores *z_ij _*so that for each gene *i *the normalized expression value has mean *μ_i _*= 0 and standard deviation *σ_i _*= 1 over sample *j*.

### Selection of protein pool and construction of network-based biomarker

To integrate the gene expression and PPI information data and construct the network-based biomarker consisting of protein association networks, the expression value of each gene was first overlaid on its corresponding protein. The gene expression for each protein was then used to select differentially expressed proteins using one-way analysis of variance (ANOVA), where the null hypothesis is that the average expression levels for the protein are the same for smokers with and without cancer. The proteins with Bonferroni adjusted *p*-values less than 0.05 were selected for the protein pool. Because we used the network-based biomarker as an investigative tool, the differentially expressed proteins without PPI information were excluded from the protein pool, while proteins based on PPI information that were highly connected with pool proteins were also included. In other words, the protein pool consisted of both differentially expressed proteins and the proteins that are highly connected with them. Based on the protein pool and PPI information, a rough PPI network can be easily constructed by linking proteins that share interactions. It is worth noting that since the data for cancer and non-cancer samples are limited, the number of proteins selected for rough PPI network construction is also restricted. That is, to avoid overfitting in network construction, the maximum degree of the proteins in the rough PPI network should be less than the cancer/non-cancer sample number, thereby restricting the size of the rough PPI network.

Using a simple regression model, the rough PPI network was further refined with the microarray data to highlight the independent protein association for samples with and without lung cancer relative to their respective data sets. For a target protein *i *in the rough PPI network, the protein was described using the following protein association model:

(1) $$y_{i}[n]=\sum_{k=1}^{N_{i}}\alpha_{ik}y_{ik}[n]+\varepsilon_{i}[n]$$

where, *y_i_*[*n*] represents the gene expression level of the target protein *i *for the sample *n; α_ik _* denotes the association ability between the target protein *i *and its *k*^-th ^interactive protein, which quantifies the expression relation between the interactive proteins and can be identified using our data; *y_ik_*[*n*] indicates the gene expression level of the *k*^-th ^protein that interacts with the target protein *i *for sample *n*; *N_i _*represents the number of proteins interacting with the target protein *i *and can be obtained from the rough PPI network; and *ε_i_*[*n*] denotes stochastic noise associated with other factors or model uncertainty. Equation (1) states that, biologically, the expression level of the target protein *i *is associated with the expression levels of interacting proteins. A protein association model was constructed for each protein in the protein pool.

After the protein association model of the rough PPI network was constructed, the association parameters in equation (1) were identified using maximum likelihood estimation method [27,28] using microarray data (see Additional file 1 for details). Since there are two data sets of microarray data (smokers with and without cancer), the association parameters were identified separately for the cancer data set and non-cancer data set, resulting in *α*_*ik,*C _and *α*_*ik*,N_, respectively. In this case, for each protein in each phenotype, i.e., with cancer or without cancer, a mathematical description was constructed to characterize the respective expression association. Once the association parameters for all proteins in the rough PPI network were identified, the significant protein associations were determined based on the estimated association abilities (*α*_*ik*_'s). Akaike Information Criterion (AIC) [27,29] and Student's t-test [30] were employed for model order selection and for determining significance of protein associations (see Additional file 1 for details). In doing so, the rough PPI network was refined and the protein association networks for smokers with or without cancer were constructed.

Based on the identified protein association abilities, two matrices were established to represent the cancer protein association network (CPAN) and the non-cancer protein association network (NPAN).

(2) $$\begin{array}{l} C=\left[\begin{array}{cccc} \alpha_{11,\text{C}} & \alpha_{12,\text{C}} & \cdots & \alpha_{1K,\text{C}}\\ \alpha_{21,\text{C}} & \alpha_{22,\text{C}} & \cdots & \alpha_{2K,\text{C}}\\ \vdots & \vdots & \ddots & \vdots \\ \alpha_{K1,\text{C}} & \alpha_{K2,\text{C}} & \cdots & \alpha_{KK,\text{C}} \end{array}\right]\\ N=\left[\begin{array}{cccc} \alpha_{11,\text{N}} & \alpha_{12,\text{N}} & \cdots & \alpha_{1K,\text{N}}\\ \alpha_{21,\text{N}} & \alpha_{22,\text{N}} & \cdots & \alpha_{2K,\text{N}}\\ \vdots & \vdots & \ddots & \vdots \\ \alpha_{K1,\text{N}} & \alpha_{K2,\text{N}} & \cdots & \alpha_{KK,\text{N}} \end{array}\right] \end{array}$$

where, *α*_*ij*,C _and *α*_*ij*,N _indicate the quantitative protein association ability between protein *i *and protein *j *for CPAN and NPAN, respectively; and *K *is the number of proteins in the protein association network. For a given protein *i *and protein *j *in the protein association network, the association ability *α*_*ij *_quantifies the expression relation between the interactive proteins. If *α*_*ij *_= 0, there is no association between protein *i *and protein *j*. Further, we said that protein *i *is associated with protein *j *means that the expression level changes of protein *i *account for the expression level changes of protein *j *and vice versa. As a consequence, when the estimated protein association ability *α*_*ij *_≠ *α*_*ji*_, the one which has larger absolute value would be selected as the association ability between protein *i *and protein *j*, i.e., *α*_*ij *_= *α*_*ji*_. The resulting CPAN and NPAN constituted the network-based biomarker used for identifying the significant proteins in lung carcinogenesis and diagnostic evaluation.

### Determination of significant proteins in lung carcinogenesis via the network-based biomarker

According to equations (1) and (2), the protein association models for CPAN and NPAN can be represented as the following equations.

(3) $$\begin{array}{l} Y_{\text{C}}=CY_{\text{C}}+E_{\text{C}}\\ Y_{\text{N}}=NY_{\text{N}}+E_{\text{N}} \end{array}$$

where $Y_{\text{C}}={\left[\begin{array}{cccc} y_{1,\text{C}}[n] & y_{2,\text{C}}[n] & \cdots & y_{K,\text{C}}[n] \end{array}\right]}^{T}$, $Y_{\text{N}}={\left[\begin{array}{cccc} y_{1,\text{N}}[n] & y_{2,\text{N}}[n] & \cdots & y_{K,\text{N}}[n] \end{array}\right]}^{T}$ denotes the vectors of expression levels; and *E*_C_ and *E*_N_ indicate the noise vectors in cancer and non-cancer cases, respectively. A matrix indicating the difference between two protein association networks is defined as *C-N*.

(4) $$\begin{array}{c} D=\left[\begin{array}{cccc} d_{11} & d_{12} & \cdots & d_{1K}\\ d_{21} & d_{22} & \cdots & d_{2K}\\ \vdots & \vdots & \ddots & \vdots \\ d_{K1} & d_{K2} & \cdots & d_{KK} \end{array}\right]\\ =\left[\begin{array}{cccc} \alpha_{11,\text{C}}-\alpha_{11,\text{N}} & \alpha_{12,\text{C}}-\alpha_{12,\text{N}} & \cdots & \alpha_{1K,\text{C}}-\alpha_{1K,\text{N}}\\ \alpha_{21,\text{C}}-\alpha_{21,\text{N}} & \alpha_{22,\text{C}}-\alpha_{22,\text{N}} & \cdots & \alpha_{2K,\text{C}}-\alpha_{2K,\text{N}}\\ \vdots & \vdots & \ddots & \vdots \\ \alpha_{K1,\text{C}}-\alpha_{K1,\text{N}} & \alpha_{K2,\text{C}}-\alpha_{K2,\text{N}} & \cdots & \alpha_{KK,\text{C}}-\alpha_{KK,\text{N}} \end{array}\right] \end{array}$$

where *d_ij _*denotes the difference in protein association ability between CPAN and NPAN among protein *i *and protein *j*. Using the matrix *D *to show the difference in network structure between CPAN and NPAN, a carcinogenesis relevance value (CRV) was presented to quantify the correlation of each protein with significance of lung carcinogenesis. To identify the significant proteins in lung carcinogenesis, two important issues were taken into consideration. First, the magnitude of the association abilities *α*_*ij*_'s denotes the significance of one protein to the other one. A higher absolute value of *α*_*ij *_implies that the two proteins are more tightly associated. Second, if a protein plays a more crucial role in lung carcinogenesis, the difference in association numbers linked to the protein for CPAN and NPAN would be larger. For example, if one protein shares a strong association with many proteins in CPAN, but a weaker association (no protein) in NPAN, the protein in question is more likely involved in lung carcinogenesis. As a result, the CRV was determined based on the difference in protein association abilities via the following equation.

(5) $${\text{CRV}}_{i}=\sum_{j=1}^{K}\left|d_{ij}\right|$$

For the *i*^-th ^protein in the network-based biomarker, the implication of equation (5) is that the CRV quantifies the extent of protein associations that differentiate CPAN from NPAN.

**Figure 2:**
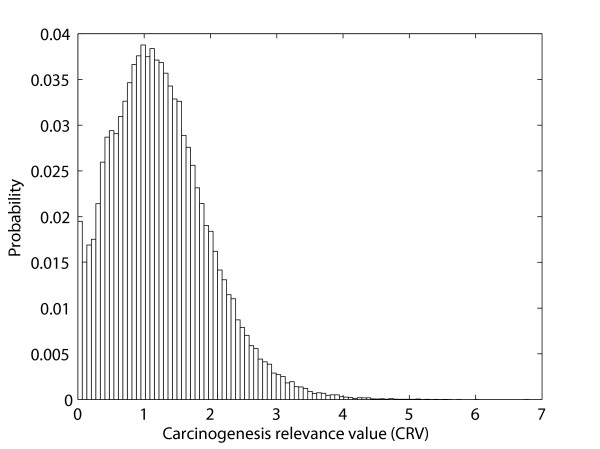
**Distribution of carcinogenesis relevance values (CRVs) of random networks**. The null distribution of CRVs is generated by 100,000 randomly permuted network structures.

In addition to the CRV assigned, an empirical *p*-value was also obtained for each protein to determine the statistical significance of the CRV. To determine the *p*-value for an observed CRV, a null distribution of CRVs (Figure 2) was generated by repeatedly permuting the network structure of the rough PPI network and computing the CRV for each random network structure. The permutation of the network structure was performed by maintaining network size, i.e., interacted proteins were permuted without altering the total number of protein interactions. The process was repeated 100,000 times and the *p*-value of the corresponding CRV was estimated as the fraction of random network structures whose CRV is at least as large as the CRV of the real network structure. The CRVs with *p*-value ≤ 0.05 were determined as significant CRVs and the corresponding proteins were identified as significant proteins in lung carcinogenesis.

### Diagnostic evaluation by the network-based biomarker

An important feature of the proposed network-based biomarker approach is its ability to identify significant proteins in lung cancer, and perform accurate diagnosis of smokers suspect of lung cancer. Using the new microarray expression data for smokers, we can classify a sample into smoker with cancer or smoker without cancer based on CPAN and NPAN within the network-based biomarker. The idea comes from the similarity comparison of new sample data between CPAN and NPAN. Specifically, sample data that are more similar to the network structure of CPAN than to NPAN signifies the presence of lung cancer in the smoker, and vice versa. Because the network construction uses more than one sample, the new sample data were mapped to the CPAN and NPAN identified above, with the mapping error employed as the criteria for classification. Assuming we had new sample data $Z={\left[\begin{array}{cccc} z_{1} & z_{2} & \cdots & z_{K} \end{array}\right]}^{T}$ from a smoker, based on equations (1) and (3), the mapping errors for CPAN and NPAN, respectively, are defined as

(6) $$\begin{array}{l} {\text{ME}}_{\text{C}}={\left\|Z-C\cdot Z\right\|}_{2}\\ {\text{ME}}_{\text{N}}={\left\|Z-N\cdot Z\right\|}_{2} \end{array}$$

where ${\left\|P\right\|}_{2}={\left(\sum_{i=1}^{K}p_{i}^{2}\right)}^{1/2}$ when $P={\left[\begin{array}{cccc} p_{1} & p_{2} & \cdots & p_{K} \end{array}\right]}^{T}$. The mapping errors can be considered as the similarity measurement of the new sample *Z *to the CPAN and NPAN systems. The smaller the mapping error, the stronger the correlation between the sample data and the protein association network. Consequently, if ME_C _< ME_N_, the new sample *Z *is more similar to the cancer system and is categorized into smokers with cancer, and vice versa. The criteria of mapping errors have simultaneously taken into account the protein association network structures with quantitative association abilities and the expression levels of the proteins. Further, since the modeling error operates as the criterion of classification, the classification is more dependent on network structure than data alone and thus could also be suitable for classification using independent data. We believe that this kind of classification approach can provide new perspective for diagnostic evaluation.

## Results

### Construction of network-based biomarker and identification of significant proteins in lung carcinogenesis

**Figure 3:**
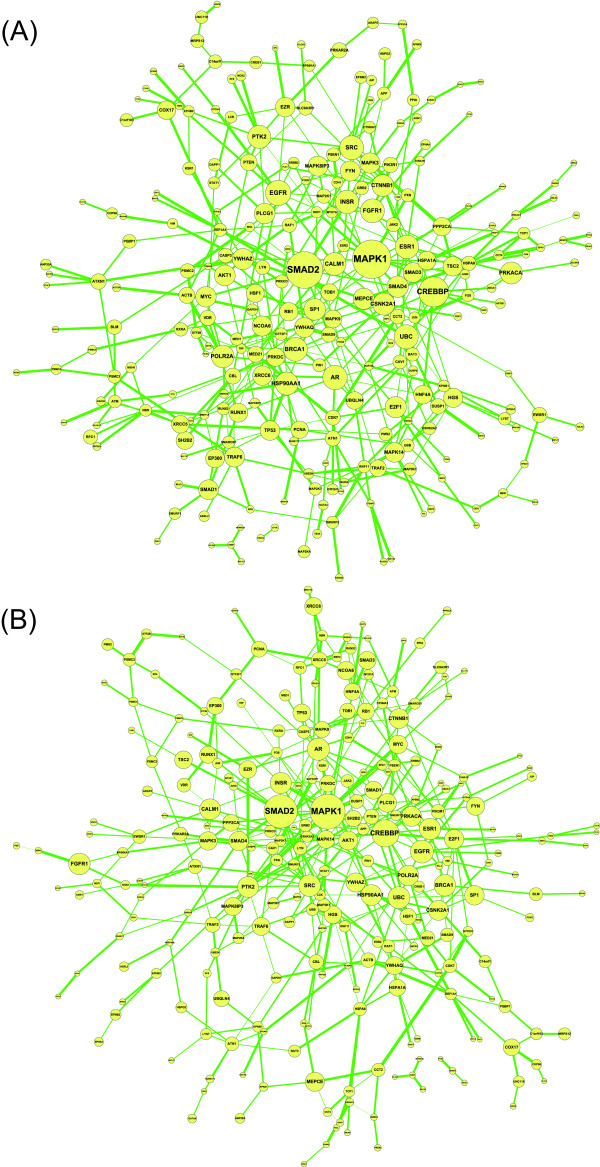
**The constructed network-based biomarker (A) Cancer protein association network (CPAN) (B) Non-cancer protein association network (NPAN)**. The node size is proportional to the CRV for each protein and the edge width represents the magnitude of the association ability between the two proteins. The figures are created using Cytoscape [77].

**Figure 4:**
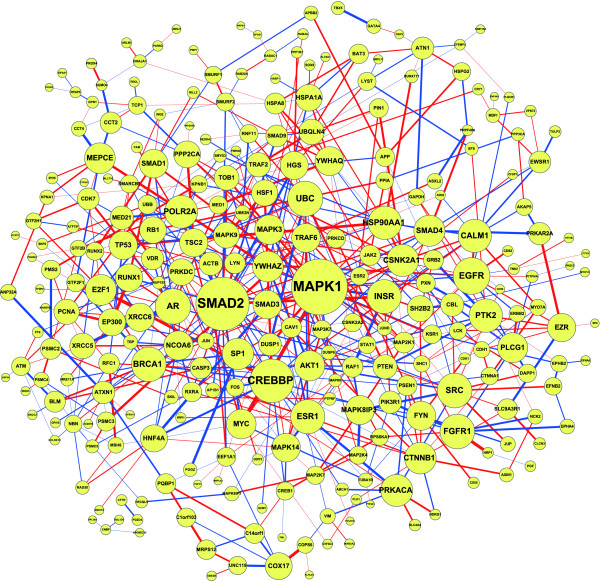
**The difference between CPAN and NPAN**. The node size is proportional to the CRV for each protein and the edge width represents the magnitude of the association ability between the two proteins. Red and blue edges indicate the positive and negative values of *d_ij_*'s in equation (4), respectively. The figure is created using Cytoscape [77].

We applied the proposed network-based biomarker approach for molecular investigation and diagnosis of lung cancer. The primary data set (79 smokers with lung cancer and 73 smokers without lung cancer) of GSE4115 downloaded from the GEO database http://www.ncbi.nlm.nih.gov/geo/ was used for construction of the network-based biomarker. Based on ANOVA, 199 proteins with PPI information were identified as the differentially expressed proteins and were selected in the protein pool. In addition, the proteins that linked to three differentially expressed proteins in the protein pool according to PPI information were also included in the pool. As a result, the protein pool consisted of 339 proteins. Following this, proteins with PPI information among them were linked together, resulting in the rough PPI network. The expression profiles for smokers with or without cancer and the protein association model (1) were further employed to refine the rough PPI network. The CPAN and NPAN, which consisted of 399 and 393 protein associations respectively, would constitute the network-based biomarker of lung cancer (Figure 3). The difference between CPAN and NPAN is expressed further in Figure 4. According to the CPAN and NPAN with quantitative association abilities, the CRVs for each protein were computed and the significance of these CRVs was determined. Consequently, 40 identified proteins played significant roles in lung carcinogenesis (Table 1 and Additional file 2).

**Table 1:** The identified significant proteins in lung carcinogenesis

Protein symbol^†^	CRV	*p*-value	Functional annotation*			Literature evidence^§^
			Cell growth	Cell survival	Cell migration	
MAPK1	8.3418	< 1e-5	+	+	+	[31,32]
SMAD2	7.7901	< 1e-5	+	+	+	[36]
CREBBP	5.7870	0.00002	+			[44]
EGFR	4.3635	0.00086	+	+	+	[39-41]
AR	4.0966	0.00159	+	+	+	[49]
UBC	4.0331	0.00180				
SRC	3.9446	0.00218	+	+	+	[51,63]
FGFR1	3.9227	0.00237	+		+	[42]
BRCA1	3.9049	0.00243	+	+		[58]
ESR1	3.8409	0.00295	+	+	+	[50]
INSR	3.7946	0.00329	+		+	[43]
PTK2	3.6758	0.00432	+	+	+	[63,64]
HSP90AA1	3.6732	0.00436	+	+	+	[54]
CALM1	3.6363	0.00482		+		
POLR2A	3.5701	0.00547				
CSNK2A1	3.4128	0.00761	+	+		[61]
PRKACA	3.3688	0.00856		+		
CTNNB1	3.2935	0.00994	+	+	+	[45]
SP1	3.2397	0.01133	+	+		[59]
SMAD4	3.1947	0.01266	+	+	+	[36]
E2F1	3.1382	0.01407	+	+		[46]
YWHAZ	3.1212	0.01467	+			[52]
MEPCE	3.0968	0.01545				
AKT1	3.0193	0.01857	+	+	+	[62]
PLCG1	2.9654	0.02069			+	[66]
MYC	2.8987	0.02385	+	+		[47]
MAPK3	2.8545	0.02654	+	+	+	[31,32]
NCOA6	2.8132	0.02892	+	+		
FYN	2.7833	0.03089	+		+	[48]
MAPK8IP3	2.7746	0.03141			+	
YWHAQ	2.7582	0.03242	+			[53]
TRAF6	2.7150	0.03535		+		[57]
SMAD1	2.6940	0.03697	+	+	+	[37]
SMAD3	2.6815	0.03815	+	+	+	[36]
MAPK14	2.6727	0.03894	+	+	+	[34]
TP53	2.6522	0.04056	+	+	+	[40,41,55]
XRCC6	2.6270	0.04263		+		
EZR	2.6213	0.04314			+	[67]
TSC2	2.6116	0.04401	+	+	+	[65]
HGS	2.5730	0.04744	+			

^† ^The full names of these proteins according to UniProt database http://www.uniprot.org/ are listed in Additional file 2.

* The functional annotations are from the Gene Ontology database http://www.geneontology.org/ and literatures.

^§ ^The literature evidences indicate that overexpression/dysregulation of the specific protein or mutation of the corresponding gene would result in carcinogenesis.

### Investigation into the significant proteins in lung carcinogenesis

**Figure 5:**
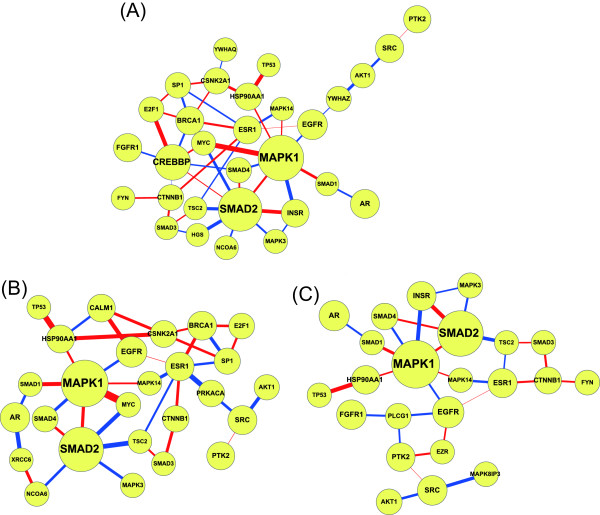
**The functional subnetworks of significant proteins identified according to the network-based biomarker (A) Cell growth functional subnetwork (B) Cell survival functional subnetwork (C) Cell migration functional subnetwork**. All the functional subnetworks are extracted from Figure 4.

The 40 identified proteins contributing to lung carcinogenesis can be divided into three categories according to the functional annotations (Table 1); the three functional subnetworks are shown in Figure 5. The mechanisms in carcinogenesis using the significant proteins were further investigated through various cell life cycle stages.

#### (1) Cell growth

Cancer is a complex disease of uncontrolled cell growth. Therefore, the proteins responsible for cell growth are likely to play critical roles in lung carcinogenesis. Among the 40 significant proteins identified based on the network-based biomarker, 30 proteins are annotated with cell growth (Table 1). The mitogen-activated protein kinase (MAPK) cascade is a highly conserved module that is identifiable in many cancers. Three MAPK-related proteins annotated with cell growth, MAPK1 (ERK2), MAPK3 (ERK1) and MAPK14 (p38α), were identified as significant proteins in lung carcinogenesis. Elevated expression of activated MAPK1/3 have been observed in NSCLC [31] and may play a role in lung metastasis [32]. MAPK14 (p38α) is well known for its important role in response to inflammation and environmental stress [33]; its protein expression is more than three times lower in human lung tumors compared with normal human lung tissue, suggesting that MAPK14 may function as a negative regulator of lung carcinogenesis [34]. Four proteins from the SMAD protein family (SMAD1, SMAD2, SMAD3 and SMAD4) were also identified as significant proteins in lung carcinogenesis. The SMAD proteins, which consist of three functional classes, are signal transducers and transcriptional modulators [35]. Mutations and altered expression for these four proteins were observed in human cancer [36,37], highlighting the significant, albeit varied, roles of the SMAD protein family in carcinogenesis.

EGFR, FGFR1, and INSR are receptor tyrosine kinases (RTKs) bound by an epidermal growth factor, a fibroblast growth factor, and insulin, respectively. Receptor tyrosine kinases have been shown to not only perform a key regulatory role in normal cellular processes, but also become critically involved in the development and progression of human cancers [38]. EGFR is one of the most extensively studied proteins in carcinogenesis. It is overexpressed in NSCLC as well as in other common tumors, and its increased expression is associated with aggressive tumor growth and therapy resistance [39]. EGFR has also been found to be significantly mutated in lung adenocarcinoma [40,41]. Because of its significance, EGFR becomes a popular therapeutic target for carcinogenesis. Gefitinib (Iressa) and erlotinib (Tarceva) are two targeted therapies that specifically inhibit EGFR tyrosine kinase [39,41]. FGFR1 and INSR are both known to be involved in carcinogenesis and thus become novel, attractive targets for cancer therapeutic strategies [42,43].

Many proteins acting as transcriptional regulators were also identified. CREBBP is a transcriptional co-activator downstream of the TGFβ pathway and the mutations and deletions of the CREBBP gene are associated with lung cancer [44]. CTNNB1 (β-catenin) is one of the core components in the Wnt pathway. Mutation of β-catenin, which results in aberrant activation of the Wnt pathway, is a frequent cause of human cancer growth [45]. E2F1 is one of the significant proteins involved in the cell cycle. Its overexpression has been demonstrated in both NSCLC and SCLC and is induced by its upstream RB protein [46]. MYC is a nuclear phosphoprotein and functions as a transcription factor. It controls cell cycle progression by simulating G1/S transition and may result in loss of cell cycle arrest and uncontrolled tumor growth when dysfunctional [47]. Other identified significant proteins such as AR, ESR1, SRC, FYN, YWHAQ, YWHAZ, and HSP90AA1 were also shown to contribute to the process of carcinogenesis [48-54].

#### (2) Cell survival

The ability of tumor cell populations to expand in number is determined not only by the rate of cell proliferation but also by the rate of cell death [13]. In addition, the acquired resistance of programmed cell death, apoptosis, is a hallmark of cancer. Consequently, proteins annotated with cell survival might be important in carcinogenesis. Twenty-seven significant proteins identified by the network-based biomarker were annotated with cell survival. TP53 (p53) is a well-studied tumor suppressor protein and plays important roles in anti-cancer mechanisms. Its activation is induced by a number of stress signals such as DNA damage, oxidative stress and activated oncogenes. Activated p53 induces cell cycle arrest, apoptosis and inhibition of angiogenesis and metastasis. Once damaged, tumor suppression is severely reduced, resulting in uncontrolled proliferation of the cell. Due to the importance in carcinogenesis, it is no surprise that p53 was found to be significantly mutated in lung adenocarcinoma as well as in squamous cell carcinoma and SCLC [40,41,55].

TRAF6 functions as a signal transducer in the NFκB pathway that activates IKK, in response to proinflammatory cytokines. The identification of TRAF6 by the proposed network-based biomarker approach reinforces the linkage between inflammation and cancer [56,57]. BRCA1 is a nuclear phosphoprotein that contributes to genomic stability. The mutant phenotype of BRCA1 predisposes to breast and ovarian cancer [58]. SP1 is a transcription factor downstream of the TGFβ pathway and its overexpression contributes to malignant transformation [59]. Other protein kinases, AKT and CSNK2A1 (CK2), were also shown to participate in the carcinogenic process [60-62].

#### (3) Cell migration

With the progression of cancer, the malignant tumor cells acquire the ability to migrate and metastasize to distant sites. As a result, the proteins that are relevant to the cell migration capability are crucial for the carcinogenic process. Twenty-three out of 40 significant proteins were annotated with cell migration. PTK2 (FAK), a protein tyrosine kinase in the RTK pathway, is an important mediator within the cell migration process, as well as in cell proliferation and cell survival. Substantial evidence has shown that activated PTK2 leads to tumor growth and metastasis [63], and the level of expression is substantially linked to the invasive potential of tumors [64]. High levels of TSC2 were correlated with increased metastasis and reduced survival in breast cancer patients, revealing a protumorigenic role for TSC2 [65]. The other two significant proteins, PLCG1 and EZR (ezrin), were demonstrated to play critical roles in the metastatic potential of cancer cells but not in primary tumor growth [66,67].

For nine out of the 40 significant proteins identified, little is known about their roles in lung cancer (see Table 1). UBC is identified as a polyubiquitin precursor. Protein ubiquitination is a fundamental, regulatory post-translational modification controlling intracellular signaling events. It has been associated with protein degradation, DNA repair, cell cycle regulation, endocytosis, and kinase modification [68]. Dysregulation of ubiquitin-mediated signaling is increasingly implicated in some human diseases. Therefore, UBC may be an important target for further characterization of lung carcinogenesis. CALM1 is calmodulin, which mediates the control of a large number of enzymes and other proteins by Ca^2+^. It is an essential regulator of cell cycle progression and cell survival. Further research is needed to examine its relation with carcinogenesis. PRKACA is a cAMP-dependent protein kinase. The identification of PRKACA as a significant protein implies that cAMP signaling might also be involved in lung carcinogenesis. MAPK8IP3 functions as a scaffold protein in the RTK pathway; NCOA6, HGS, and XRCC6 are annotated with cell growth and/or cell survival. However, until now, no empirical evidence has linked their relevance to carcinogenesis, which makes them potential targets for further investigation into lung carcinogenesis.

### Diagnostic evaluation of smokers suspect of lung cancer using the network-based biomarker

The network-based biomarker was constructed based on the primary data set of GSE4115. An independent data set (the prospective data set of GSE4115, 18 smokers with lung cancer and 17 smokers without lung cancer) was then used to evaluate the diagnostic performance of the proposed network-based biomarker. Among the 35 samples, 26 were accurately classified, resulting in an accuracy of 74.29%. The sensitivity and specificity of the proposed approach were also evaluated. The network-based molecular diagnosis can identify smokers with or without cancer, using a high level of sensitivity of 83.33%, and a moderate specificity of 64.71%: this enables the proposed network-based biomarker to effectively diagnose the smokers with lung cancer. Further, this approach enables the network-based biomarker to act as a screening test, which, with the aid of other clinical diagnostic tools, both accelerates the process and improves the sensitivity of diagnosis.

The cause of the moderate specificity was further investigated. The difference between the specificity value and the sensitivity value can be attributed to a misclassification of a number of smokers without cancer in the cancer category. The misclassification may be due to similarities in molecular pattern, i.e., the gene expression profiles of smokers with cancer are similar to those of smokers without cancer. In order to validate the hypothesis, Pearson correlation coefficients [30] of gene expression profiles for both smokers with and without cancer were calculated. The mean correlation coefficient of smokers with cancer was 0.9616, whereas the mean correlation coefficient of smokers without cancer was 0.9441. In addition, the mean correlation coefficient of smokers both with and without cancer (pooled) was as high as 0.9437, suggesting that the molecular patterns shared among smokers with and without cancer are indeed highly similar. Because of the highly similar molecular patterns and the fact that cigarette smoking is the main instigator of lung cancer, it is likely smokers without cancer could one day develop lung cancer.

In order to validate the predictive performance of the proposed network-based biomarker, several comparisons were made. First, we tested the predictive performance without the information of protein-protein interactions. The 199 differentially expressed proteins selected by ANOVA using the primary data set were used for classification of the prospective data set. A simple hierarchical clustering was performed (see Additional file 3), illustrating that the use of gene expression alone cannot accurately classify the prospective data set (65.71% accuracy). The comparison shows that the integration of gene expression profiles and protein interaction information can improve lung cancer diagnosis. Second, we compared the predictive performance of the proposed method with randomly selected networks. The average accuracy for 100,000 randomly selected networks of 339 proteins was 48.44%, highlighting the significance of the proposed network-based biomarker. Third, in addition to evaluating the predictive performance using an independent data set, a 5-fold cross-validation was applied to the primary data set plus the prospective data set. The accuracy of the cross-validation is similar to the initial accuracy of 74.29% computed using the independent data set, illustrating the robustness and reproducibility of the proposed network-based biomarker approach.

## Discussion

Cancer is a complex disease and carcinogenesis in humans is a multistep process that transforms normal cells into malignant derivatives. Many researchers are investigating the underlying mechanisms that prompt the uncontrolled cell proliferation and metastasis. They have successfully identified some key components of the various steps in the carcinogenesis and some therapeutic interventions have been developed to at least slow down the carcinogenic process. However, because of the complexity, the therapy that targets some specific molecules is only partially effective and tumor-specific. Therefore, investigation of the carcinogenesis from the systems perspective is inevitable. On the other hand, biomarker identification for cancer diagnosis has been a primary research focus in the biomedical field since the use of biomarkers could provide early detection of cancer. As a result, in this study, a network-based biomarker approach has been proposed to simultaneously account for molecular investigation and diagnosis. The proposed approach was applied on the sample data obtained from smokers with and without lung cancer and 40 significant proteins were identified in lung carcinogenesis. The network-based biomarker considers not only differentially expressed proteins but also the protein association network structure. This allows an accurate identification of proteins with low discriminative potentials if such proteins were associated with many other significant proteins [15]. This property is important for the identification of significant proteins in lung carcinogenesis and provides a mechanistic insight into the process. From the mechanism investigation of the 40 significant proteins identified using the network-based biomarker, we found that the significant proteins identified are involved in the pathways that are responsible for cellular processes, including proliferation, differentiation, apoptosis, and metastasis. More importantly, from the results presented, we found that dysregulated signals exist in multiple pathways. There are two possible explanations for the result: the genetic mutations are accumulated respectively to components of different pathways, or the aberrant signals affect different pathways through cross-talk mechanisms. Further investigation is needed to address these two hypotheses. In addition to the investigation of significant proteins, the network-based biomarker can be used as a type of screening test with high sensitivity. Using the same data (the prospective data set of GSE4115), conventional bronchoscopy was shown to be 44% sensitive to cancer detection [3], which is only half of the proposed network-based biomarker. This further reinforces the clinical utility of the network-based biomarker.

Although our proposed method is shown to be useful, some limitations exist and the need for further improvements remains. In the proposed network-based biomarker approach, gene expression profiles were overlaid to the corresponding proteins for further analysis. However, levels of mRNA do not always correlate with protein levels and do not provide information on post-translational modification such as phosphorylation that may be critical in regulating protein activity [41]. Consequently, emerging high-throughput proteomic techniques such as protein microarrays would benefit our method by significantly improving the detection performance over mRNA microarray data. In addition, if the genome-wide gene expression levels and protein expression levels can be obtained simultaneously, we are then able to construct the integrated cellular networks of transcription regulations and protein interactions which provide a more integrated network-based biomarker [69]. The protein-protein interaction data from public databases also plays important roles in the proposed method. Nevertheless, there is a large variation in the coverage of protein interaction data across the interaction databases [70]. Therefore, HPRD and BioGRID databases were integrated for the PPI information in this study. We believe that the increased quality and coverage of protein interaction data would enhance the proposed network-based biomarker approach for characterization of lung carcinogenesis. Another limitation of the proposed method was the restriction in the size of the protein association network from the sample size available due to the avoidance of overfitting in the network construction. This results in the exclusion of some well-studied proteins that are relevant to the lung carcinogenic process in the network-based biomarker, including KRAS, MET, PI3KCA. To overcome the problem, more samples are needed. We believe that improvements to diagnostic evaluation using the network-based biomarker lie in the expansion of the constructed protein association networks. Many other groups identified the discriminative subnetworks using different methods, especially graph theory-based methods [71,72]. For example, Tian *et al*. proposed a hypergraph-based iterative learning algorithm for subnetwork identification, which minimizes a cost function under a unified regularization framework [72]. These graph-based methods can also be incorporated to improve the significant protein selection in the proposed method. Further, in this study, the samples for gene expression profiling are simply divided into two groups: smokers with cancer and without cancer. With more sample data, particularly cancer stage-specific samples, we can determine how the network evolves and changes during cancer progression using the proposed method.

Our network-based biomarker provides both a systematic insight into the lung carcinogenic process and a good method for identifying significant proteins, categorized as lung cancer-related proteins and many others that have not been previously reported. These proteins not only provide new targets for further research into understanding the mechanisms of lung carcinogenesis, but are also potential targets for therapeutic interventions. The main challenge of cancer research is to find an effective therapeutic approach that specifically kills malignant cells. Conventional chemotherapy acts by killing all rapidly dividing cells, resulting in toxic effects and damage to normal tissues [73]. With the advances in understanding the mechanisms of the carcinogenic process, the so-called targeted therapy, which is more effective and less harmful to normal cells, is developed to inhibit the specific molecules that play crucial roles in tumor growth. The significant proteins identified by the proposed network-based biomarker provide suitable molecules to be targeted. For example, gefitinib (Iressa) and erlotinib (Tarceva) are two tyrosine kinase inhibitors that specifically target EGFR. Despite their effectiveness, there are still patients that do not respond well to these drugs [74]. One explanation is that single-target agents are likely to result in network compensation and drug resistance [75]. As a result, multi-target therapeutic interventions that affect multiple targets simultaneously may be required for effective control against cancer. Because biological systems are unable to perform optimally under the influence of two or more simultaneously administered drugs, multi-target therapeutics can prove effective as they may be less vulnerable to adaptive resistance from the human body [76]. With the help of significant proteins identified by the proposed network-based biomarker approach and the pathway information, it is possible the multi-target therapeutic interventions that act on different critical pathways in lung carcinogenesis can be developed.

## Conclusions

Lung cancer is the leading cause of cancer deaths worldwide. Understanding the causes and the underlying mechanisms can help fight the disease. In this study, a network-based biomarker approach, which integrated gene expression profiles and protein interaction information, was developed for molecular investigation and diagnosis for lung cancer. From a systems perspective, the constructed network-based biomarker further evaluated the lung carcinogenic process by use of significant protein identification and diagnostic evaluation. The diagnostic results indicate that the network-based biomarker is sensitive to the diagnosis of smokers with lung cancer and can be used as one kind of screening test. More importantly, the significant proteins identified by the network-based biomarker give mechanistic insights into the carcinogenic process and provide potential therapeutic targets to combat cancer.

## Competing interests

The authors declare that they have no competing interests.

## Authors' contributions

YCW developed the method, performed the analysis, evaluated the results and wrote the manuscript. BSC provided essential guidance and revised the manuscript. All authors read and approved the final manuscript.

## Pre-publication history

The pre-publication history for this paper can be accessed here:

http://www.biomedcentral.com/1755-8794/4/2/prepub

## Supplementary Material

Additional file 1 **Supplementary Methods**. Click here for file

Additional file 2 **The full names of the significant proteins identified**. Click here for file

Additional file 3 **The hierarchical clustering for 199 differentially expressed proteins of the prospective data set**. Click here for file
