# Supplementary material for: A network-based biomarker approach for molecular investigation and diagnosis of lung cancer
Source: BMC Med Genomics. 2011 Jan 6;4:2. doi: 10.1186/1755-8794-4-2 (PMC3027087; doi:10.1186/1755-8794-4-2)
Supplement: Additional file 1 — Supplementary Methods. [file 1755-8794-4-2-S1.PDF]

Supplementary Methods for
A network-based biomarker approach for molecular investigation and diagnosis of lung cancer

Identification of association parameters

After the protein association model of the rough PPI network was constructed, the association parameters in equation (1) were identified using maximum likelihood estimation method [1]. Equation (1) can be written in the following regression form.

$$\begin{aligned} y_i[n] &= \begin{bmatrix} y_{i1}[n] & \cdots & y_{iN_i}[n] \end{bmatrix} \begin{bmatrix} \alpha_{i1} \\ \vdots \\ \alpha_{iN_i} \end{bmatrix} + \varepsilon_i[n] \\ &= \phi_i[n] \cdot \theta_i + \varepsilon_i[n] \end{aligned} \quad \text{(S1)}$$

where $\phi_i[n]$ denotes the regression vector which can be obtained from the processing above, θ_i is the parameter vector to be estimated. Suppose that there are M samples for us to estimate the association parameters, $\{y_i[n] \quad \phi_i[n]\}$ for $n \in \{1, 2, \cdots, M\}$ can be easily acquired via the same procedure. In this case, equation (S1) for different samples can be represented as the following form.

$$\begin{bmatrix} y_i[1] \\ \vdots \\ y_i[M] \end{bmatrix} = \begin{bmatrix} \phi_i[1] \\ \vdots \\ \phi_i[M] \end{bmatrix} \cdot \theta_i + \begin{bmatrix} \varepsilon_i[1] \\ \vdots \\ \varepsilon_i[M] \end{bmatrix} \quad \text{(S2)}$$

For simplicity, the notations Y_i, Φ_i, and e_i were defined to express equation (S2) as follows

$$Y_i = \Phi_i \cdot \theta_i + e_i \quad \text{(S3)}$$

In equation (S2), we assumed noises $\varepsilon_i[n]$ for different samples as independent random variables of normal distribution with zero mean and unknown variance σ_i^2,

i.e., the variance of e_i is $\Sigma_i = E\left\{e_i e_i^T\right\} = \sigma_i^2 I$, where I is the identity matrix. The probability density function of e_i is given as

$$p(e_i) = \left((2\pi)^M \det\Sigma_i\right)^{-1/2} \exp\left\{-\frac{1}{2} e_i^T \Sigma_i^{-1} e_i\right\} \tag{S4}$$

Considering equations (S3) and (S4), the likelihood function can be expressed as

$$L(\theta_i, \sigma_i^2) = p(\theta_i, \sigma_i^2) = \left(2\pi\sigma_i^2\right)^{-M/2} \exp\left\{-\frac{1}{2\sigma_i^2}(Y_i - \Phi_i\theta_i)^T (Y_i - \Phi_i\theta_i)\right\} \tag{S5}$$

Maximum likelihood estimation method aims at finding θ_i and σ_i^2 to maximize the likelihood function in equation (S5). For the simplicity of computation, it is practical to take the logarithm of the likelihood function, and we have the following log-likelihood function

$$\log L(\theta_i, \sigma_i^2) = -\frac{M}{2}\log\left(2\pi\sigma_i^2\right) - \frac{1}{2\sigma_i^2}\sum_{n=1}^{M}\left[y_i[n] - \phi_i[n]\cdot\theta_i\right]^2 \tag{S6}$$

where $y_i[n]$ and $\phi_i[n]$ are the n-th element of Y_i and Φ_i, respectively. Here, the log-likelihood function is expected to have the maximum at $\theta_i = \hat{\theta}_i$ and $\sigma_i^2 = \hat{\sigma}_i^2$. The necessary conditions for determining the maximum likelihood estimates $\hat{\theta}_i$ and $\hat{\sigma}_i^2$ must conform to the following two equations.

$$\left.\frac{\partial \log L(\theta_i, \sigma_i^2)}{\partial \theta_i}\right|_{\theta_i = \hat{\theta}_i} = 0$$
$$\left.\frac{\partial \log L(\theta_i, \sigma_i^2)}{\partial \sigma_i^2}\right|_{\sigma_i^2 = \hat{\sigma}_i^2} = 0 \tag{S7}$$

After some computational deduction, the estimated parameters $\hat{\theta}_i$ and $\hat{\sigma}_i^2$ are

$$\hat{\theta}_i = \left(\Phi_i^T \Phi_i\right)^{-1} \Phi_i^T Y_i \tag{S8}$$

$$\hat{\sigma}_i^2 = \frac{1}{M}\sum_{n=1}^{M}\left[y_i[n] - \phi_i[n]\cdot\hat{\theta}_i\right]^2 = \frac{1}{M}(Y_i - \Phi_i\theta_i)^T (Y_i - \Phi_i\theta_i) \tag{S9}$$

Determination of significant protein associations

When the association parameters were identified, Akaike Information Criterion (AIC) [1,2] and Student's t-test [3], which is used to calculate the p-values of the association abilities, were employed for both model order selection and determination of significant protein associations. The AIC, which attempts to include both the estimated residual variance and model complexity in one statistics, decreases as the residual variance decreases and increases as the number of parameters increases. As the expected residual variance decreases with increasing parameter numbers for nonadequate model complexities, there should be a minimum around the correct parameter number [1,2]. Therefore, AIC can be used to select model structure based on the association abilities (α_{ik}'s) identified above. Due to computation efficiency, it is impractical to compute the AIC statistics for all possible regression models. Stepwise methods such as forward selection method and backward elimination method are developed to avoid the complexity of exhausted search [3-5]. However, in the case of backward selection method, a variable once eliminated can never be reintroduced into the model, and in the case of forward selection, once included can never be removed [3,5]. Thus, the stepwise regression method which combines forward selection method and backward elimination method was applied to compute the AIC statistics. Once the estimated regulatory parameters were examined using the AIC model selection criteria, the Student's t-test was employed to calculate the p-values for the association abilities (α_{ik}'s) under the null hypothesis $H_0 : \alpha_{ik} = 0$ [3] to determine the significant protein associations. The p-values computed were then adjusted by Bonferroni correction to avoid a lot of spurious positives [3]. The associations which adjusted p-value$\leq$0.05 were determined as significant associations and be preserved in the protein association network.

References

1. Johansson R: *System modeling and identification.* Englewood Cliffs, NJ: Prentice Hall; 1993.
2. Akaike H: **A new look at the statistical model identification.** *IEEE Transactions on Automatic Control* 1974, **19:**716-723.
3. Pagano M, Gauvreau K: *Principles of biostatistics.* 2nd edn. Pacific Grove, CA: Duxbury; 2000.
4. Hocking RR: **A Biometrics invited paper. The analysis and selection of variables in linear regression.** *Biometrics* 1976, **32:**1-49.
5. Seber GAF, Lee AJ: *Linear regression analysis.* 2nd edn. Hoboken, N.J.: Wiley-Interscience; 2003.
