# Supplementary material for: A network-based biomarker approach for molecular investigation and diagnosis of lung cancer
Source: BMC Med Genomics. 2011 Jan 6;4:2. doi: 10.1186/1755-8794-4-2 (PMC3027087; doi:10.1186/1755-8794-4-2)
Supplement: Additional file 2 — The full names of the significant proteins identified. [file 1755-8794-4-2-S2.PDF]

Supplementary Data for
A network-based biomarker approach for molecular investigation and diagnosis of lung cancer

The full names of the significant proteins identified

MAPK1: Mitogen-activated protein kinase 1
SMAD2: Mothers against decapentaplegic homolog 2
CREBBP: CREB-binding protein
EGFR: Epidermal growth factor receptor
AR: Androgen receptor
UBC: Polyubiquitin-C
SRC: Proto-oncogene tyrosine-protein kinase Src
FGFR1: Basic fibroblast growth factor receptor 1
BRCA1: Breast cancer type 1 susceptibility protein
ESR1: Estrogen receptor
INSR: Insulin receptor
PTK2: Focal adhesion kinase 1
HSP90AA1: Heat shock protein HSP 90-alpha
CALM1: Calmodulin
POLR2A: DNA-directed RNA polymerase II subunit RPB1
CSNK2A1: Casein kinase II subunit alpha
PRKACA: cAMP-dependent protein kinase catalytic subunit alpha
CTNNB1: Catenin beta-1
SP1: Transcription factor Sp1
SMAD4: Mothers against decapentaplegic homolog 4
E2F1: Transcription factor E2F1
YWHAZ: 14-3-3 protein zeta/delta
MEPCE: 7SK snRNA methylphosphate capping enzyme
AKT1: RAC-alpha serine/threonine-protein kinase
PLCG1: 1-phosphatidylinositol-4,5-bisphosphate phosphodiesterase gamma-1
MYC: Myc proto-oncogene protein
MAPK3: Mitogen-activated protein kinase 3
NCOA6: Nuclear receptor coactivator 6
FYN: Tyrosine-protein kinase Fyn
MAPK8IP3: C-Jun-amino-terminal kinase-interacting protein 3
YWHAQ: 14-3-3 protein theta
TRAF6: TNF receptor-associated factor 6
SMAD1: Mothers against decapentaplegic homolog 1

SMAD3: Mothers against decapentaplegic homolog 3
MAPK14: Mitogen-activated protein kinase 14
TP53: Cellular tumor antigen p53
XRCC6: X-ray repair cross-complementing protein 6
EZR: Ezrin
TSC2: Tuberin
