# Supplementary material for: A network-based biomarker approach for molecular investigation and diagnosis of lung cancer
Source: BMC Med Genomics. 2011 Jan 6;4:2. doi: 10.1186/1755-8794-4-2 (PMC3027087; doi:10.1186/1755-8794-4-2)

The hierarchical clustering for 199 differentially expressed proteins of the prospective data set

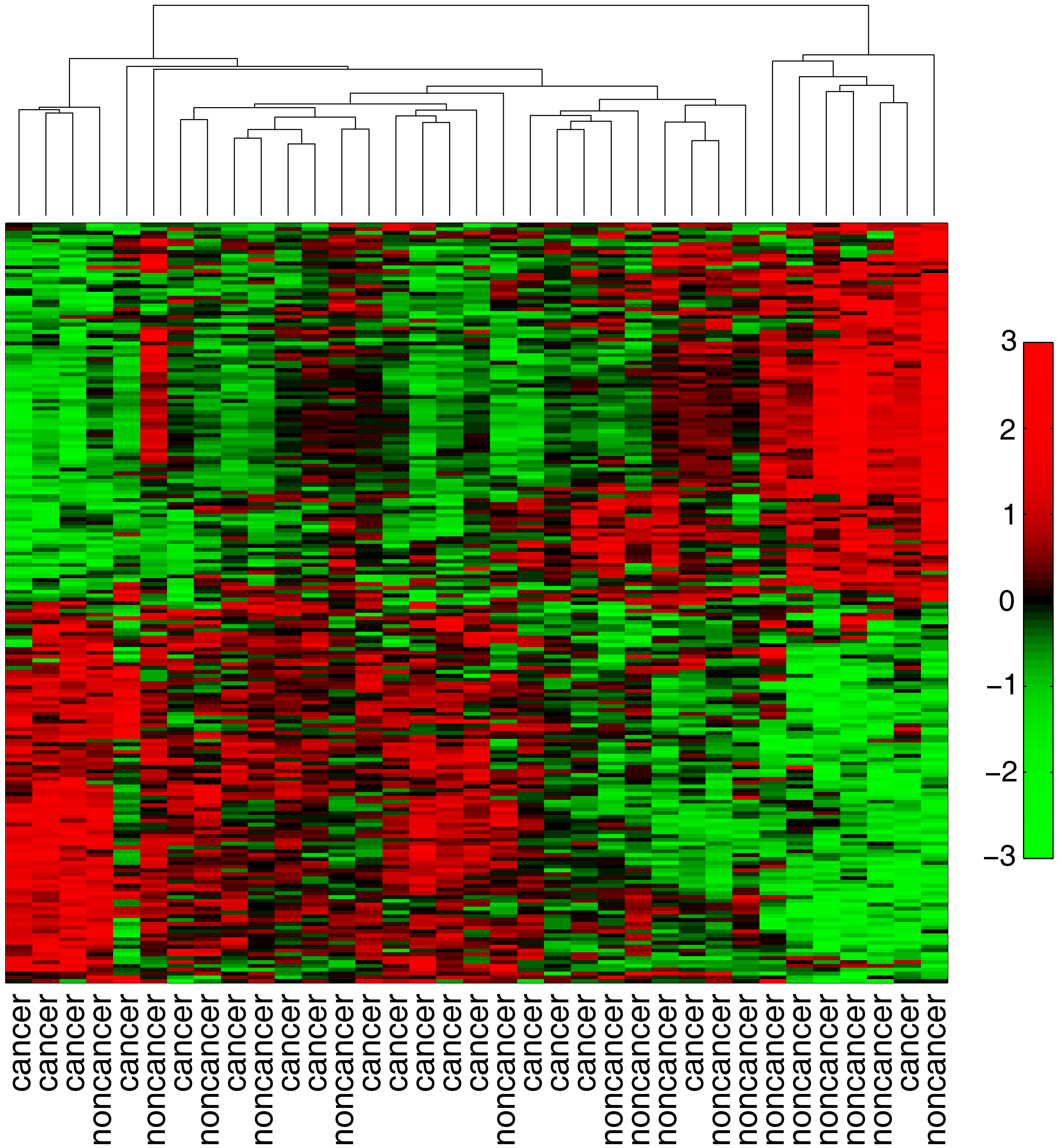

Supplement: Additional file 3 — The hierarchical clustering for 199 differentially expressed proteins of the prospective data set. [file 1755-8794-4-2-S3.PDF]
